# Neuropathologic Implication of Peripheral Neuregulin-1 and EGF Signals in Dopaminergic Dysfunction and Behavioral Deficits Relevant to Schizophrenia: Their Target Cells and Time Window

**DOI:** 10.1155/2014/697935

**Published:** 2014-05-13

**Authors:** Hiroyuki Nawa, Hidekazu Sotoyama, Yuriko Iwakura, Nobuyuki Takei, Hisaaki Namba

**Affiliations:** Department of Molecular Neurobiology, Brain Research Institute, Niigata University, 1-757 Asahimachi-dori, Chuo-ku, Niigata 951-8585, Japan

## Abstract

Neuregulin-1 and epidermal growth factor (EGF) are implicated in the pathogenesis of schizophrenia. To test the developmental hypothesis for schizophrenia, we administered these factors to rodent pups, juveniles, and adults and characterized neurobiological and behavioral consequences. These factors were also provided from their transgenes or infused into the adult brain. Here we summarize previous results from these experiments and discuss those from neuropathological aspects. In the neonatal stage but not the juvenile and adult stages, subcutaneously injected factors penetrated the blood-brain barrier and acted on brain neurons, which later resulted in persistent behavioral and dopaminergic impairments associated with schizophrenia. Neonatally EGF-treated animals exhibited persistent hyperdopaminergic abnormalities in the nigro-pallido-striatal system while neuregulin-1 treatment resulted in dopaminergic deficits in the corticolimbic dopamine system. Effects on GABAergic and glutamatergic systems were transient or limited. Even in the adult stage, intracerebral administration and transgenic expression of these factors produced similar but not identical behavioral impairments, although the effects of intracerebral administration were reversible. These findings suggest that dopaminergic development is highly vulnerable to circulating ErbB ligands in the pre- and perinatal stages. Once maldevelopment of the dopaminergic system is established during early development, dopamine-associating behavioral deficits become irreversible and manifest at postpubertal stages.

## 1. EGF-Like Ligands and Their ErbB Receptors in the Brain

**Figure 1:**
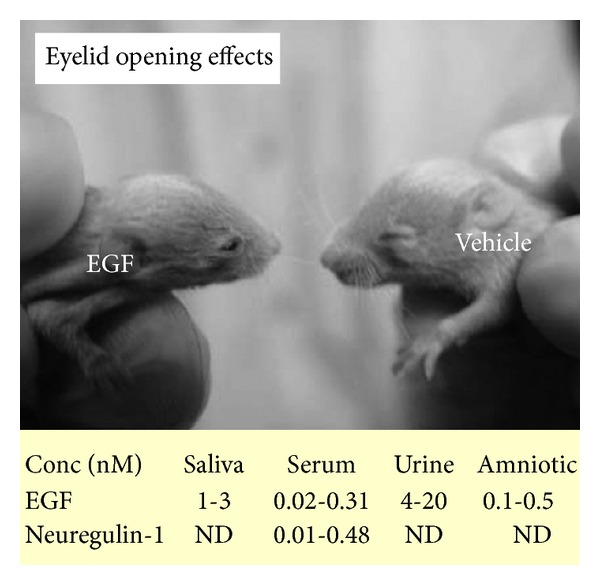
EGF and neuregulin-1 (NGR1) in body fluids mediate eyelid opening. The picture depicts the acceleration of eyelid opening following subcutaneous injections of EGF into pups. EGF (1.0 mg/kg) was subcutaneously injected to rat neonates daily from postnatal day 2 to postnatal day 10. The table shows that both EGF and NGR1 are detected in human body fluids including saliva, serum, urine, and amniotic fluids. ND: not determined.

Epidermal growth factor (EGF) was first purified from mouse salivary gland, together with nerve growth factor (NGF), and was found to induce eyelid opening activity [1] (Figure 1). Molecular cloning verified the presence of many EGF-related peptides such as heparin-binding EGF-like growth factor (HB-EGF), transforming growth factor alpha (TGF*α*), amphiregulin, and neuregulins (NRG) [2, 3]. All members of the EGF family have the capability to promote eyelid opening and are contained by various body fluids such as saliva, urine, serum, and amniotic fluids [1, 4, 5] (Figure 1). In addition to these endogenous ligands, several viruses encode EGF-like peptides in their genome and potentate host cell proliferation [6, 7]. These EGF-like peptides in blood can penetrate into the immature brain and influence neural stem cell proliferation and neuronal/glial differentiation and maturation [8–10].

**Figure 2:**
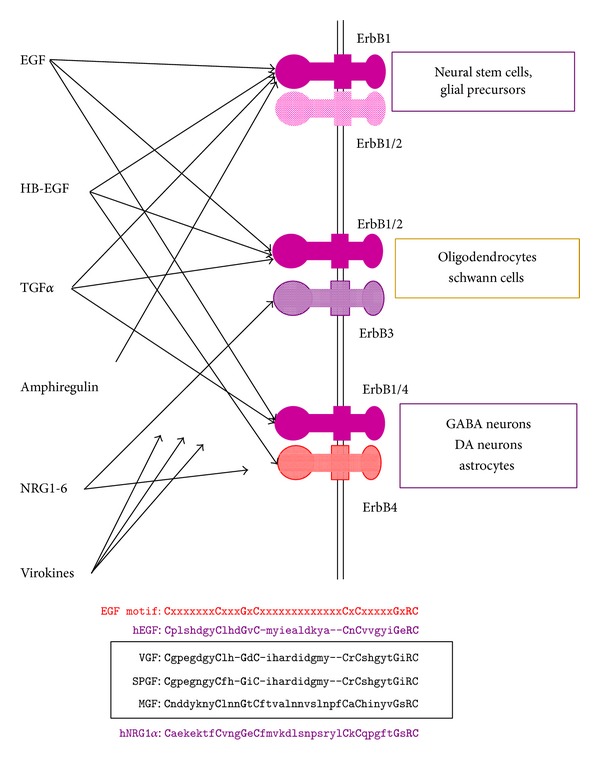
Ligands in the EGF family interact with heteromeric or homomeric ErbB receptors in GABAergic, dopaminergic (DA), and glial cells. ErbB ligands include EGF, HB-EGF (heparin-binding EGF-like growth factor), TGF*α* (transforming growth factor alpha), amphiregulin, NRG1-6, and virokines (VGF, SPGF, MGF, etc.), which associate with these ErbB receptor complexes to evoke both EGF-like and NRG-like signals. ErbB1-4 selectivity of the virokines remains to be characterized. These virokines carry the EGF-like amino acid motif common to human EGF and NRG1alpha. VGF: vaccinia virus growth factor; SPGF: smallpox virus growth factor; MGF: myxoma virus growth factor.

All members in the EGF family interact with ErbB receptor tyrosine kinases ErbB1, ErbB2, ErbB3, and ErbB4 (note: ErbB3 lacks the kinase activity). These ligands promote ErbB receptor dimer formation and transphosphorylate the partner ErbB molecule [11] (Figure 2). Each ligand has a specific binding preference with an ErbB subtype; for instance, EGF has high affinity to ErbB1, HB-EGF interacts with ErbB1 and ErbB4, and NRG1 binds to ErbB3 and ErbB4. Regardless of the ErbB subtype bound by the ligand, their signals will be transmitted from both the partner ErbB and receptor ErbB [2, 11]. Interestingly, the activated ErbB tyrosine kinase also can form a dimer with other receptor tyrosine kinases such as MET [12]. In this context, different ligands in the EGF family often evoke distinct tyrosine kinase signaling in various types of cells.

**Figure 3:**
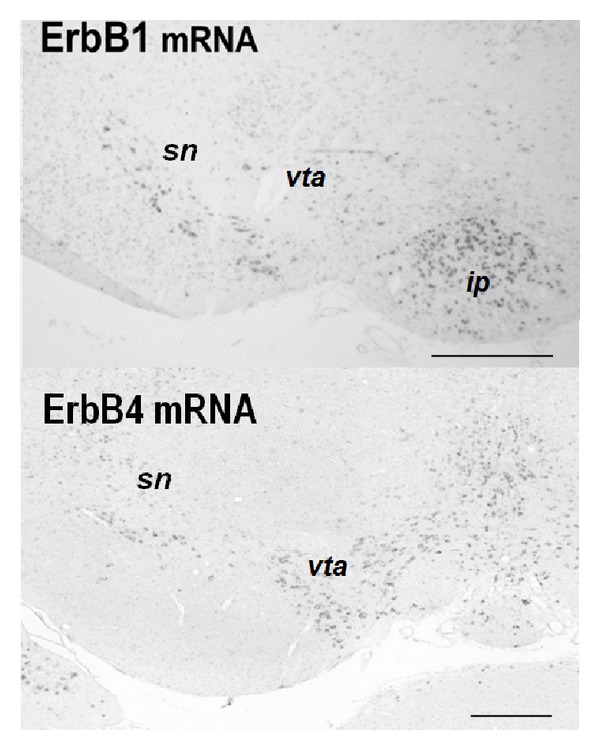
Distributions of ErbB1 mRNA and ErbB4 mRNA in rodent midbrain.* In situ* hybridization reveals enrichment of ErbB1 mRNA in the substantia nigra (sn) of rat pups (postnatal day 2). ErbB4 mRNA is expressed in both the sn and ventral tegmental area (vta) of mouse pups (postnatal day 2). Ip: interpeduncular nucleus. Scale bars = 250 *μ*m.

*In situ* hybridization techniques have revealed widespread expression of ErbB1-4 mRNAs in various types of neurons and glial cells [13–16]. EGF receptors (EGF-R or ErbB1) are highly expressed in neural stem cells. In later stages, however, lower levels of ErbB1 are also detected in GABAergic and dopaminergic neurons, which often coexpress the ErbB4 subunit as well [14, 17] (Figure 3). In contrast, the expression of ErbB3 is relatively restricted to oligodendrocytes and Schwann cells [15, 18]. The localizations of ErbB1-4 are consistent with the reports of EGF and NRG1 actions. EGF and NRG1 exert various neurotrophic activities on midbrain dopaminergic neurons [19–21] although their actions on GABAergic neurons are inconsistent among the ErbB ligands [17, 22–24].

EGF and NRG1 have been researched extensively in relation to schizophrenia. In 2002, DeCode Genetics Inc. reported a genetic association of the* NRG1* gene with schizophrenia [25] and our group found abnormal expression of EGF and ErbB1 in the postmortem brains of patients with schizophrenia [4]. Subsequently, a Finland group reported a genetic association between the* EGF* gene and schizophrenia [26–28], although this has not been replicated in all ethnic populations examined [29]. Indeed, these human studies were the impetus for our research on animal modeling of schizophrenia using EGF and NRG1.

## 2. Neurobehavioral Impact of Peripheral EGF and NRG1 Administration during Development

**Figure 4:**
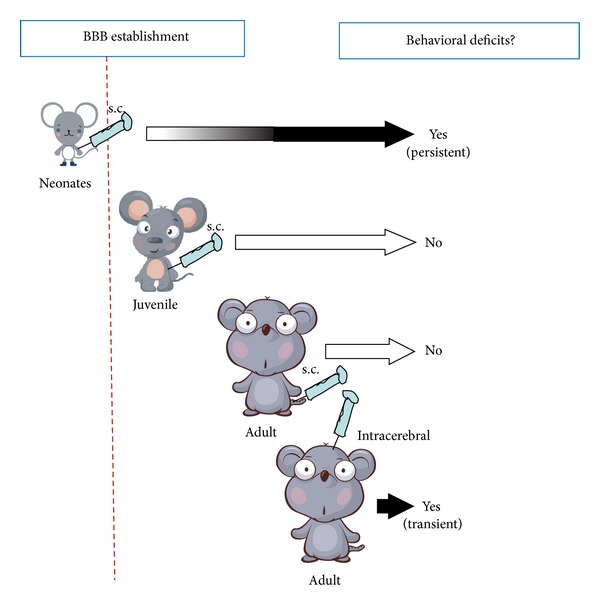
Neurobehavioral consequences following subcutaneous/intracerebral administration of EGF to neonatal, juvenile, and adult rats. Intracerebral administration to adult rats was achieved with cannula implantation to the stratum; EGF was subchronically supplied from an osmotic pump at the rate of 75 ng/h. There is a critical time window for the induction of behavioral deficits following peripheral EGF administration.

To test the contribution of these neurotrophic factors to the neurodevelopmental abnormality of schizophrenia, we subcutaneously administered the EGF protein into rats and mice at various developmental stages, neonate, juveniles, and young adults [30, 31] (Figure 4). We then monitored their behavioral traits such as prepulse inhibition scores at the adult stage. We found that neonatal exposure to EGF resulted in various behavioral deficits, most of which are implicated in schizophrenia behavioral endophenotypes. These deficits include lower prepulse inhibition, impaired latent inhibition of fear learning, reduced social behaviors, and higher sensitivity to methamphetamine and a D2 receptor agonist [30–33] (Table 1). These behavioral deficits are persistent as we have detected the prepulse inhibition deficits at postnatal month six. Although we tested more than 10 cytokines and growth factors with the above experimental procedure, EGF and NRG1 appeared to exhibit the most remarkable and persistent abnormality in behaviors [30–38].

In contrast to the neonatal injection model, the administration of EGF into the skin of juvenile or young adult rats (at the same dose) failed to induce the above behavioral abnormalities (unpublished data). Why EGF administered at the different stages has no obvious effects remains to be explored; however, it is likely to involve the differences in (1) the supply of EGF to the target (i.e., the brain permeability of EGF), (2) the sensitivity of a target to EGF (i.e., EGF receptor expression), and (3) the phenotypic nature of the reaction of the target.

Thus we first monitored the permeability of EGF through the blood-brain barrier. We found that subcutaneous injection of EGF and neuregulin-1 to rat and mouse neonates resulted in the activation (phosphorylation) of ErbB receptors in the brain and led to behavioral deficits [38] (Table 1). The receptor activation in the brain was the most remarkable at the perinatal stages and gradually diminished during the postnatal stage. After postnatal day 10, subcutaneously-injected EGF and neuregulin-1 failed to trigger marked ErbB phosphorylation in the brain [38]. When we explored the permeability of the blood-brain barrier with interleukin-1, immunohistochemistry verified the efficient diffusion of interleukin-1 from the blood vessels into the neural spaces at the rat neonatal stage but not at rat postnatal day 14 [36]. These results are in agreement with the previous microscopic observations in rats where morphological maturation of endotheliocytes and pericytes occurs on postnatal day 7 and a decrease in permeability of the hematoencephalic barrier takes place on postnatal day 10 [39]. These results suggest that the establishment of the blood-brain barrier is attained around postnatal day 10 and may be one of the key factors for determining the time dependency for the induction of behavioral impairments [36]. Even at the adult stage, however, EGF and neuregulins-1 are reported to penetrate the established blood-brain barrier in a limited degree [21, 40, 41].

The sensitivity of brain cells to EGF might also associate with the observed EGF efficacy. It is reported that ErbB1 expression gradually diminishes in the nigrostriatal system as well as in the cortical structures during postnatal development [15, 42]. This is another explanation that illustrates the developmental difference of EGF effects on animal behaviors.

We tested the first hypothesis that the penetration of the blood-brain barrier would be critical for the effectiveness of EGF injection. If it is the case, the direct EGF supply to the brain of adult rats should mimic neonatal EGF injection [43]. Indeed when EGF was subchronically infused into the striatum of adult rats from an osmotic minipump, EGF induced the deficits of prepulse inhibition and impaired latent inhibition of fear learning as was observed in the neonatal injection model. EGF infusion simultaneously elevated dopamine content and turnover as well as the enzyme activity of tyrosine hydroxylase and protein levels of dopamine transporter in the striatum, supporting its neurotrophic actions on dopamine neurons [43]. The apparent difference between the neonatal EGF model and the adult infusion model is the persistency or reversibility of the deficits. In the adult EGF-brain infusion model, the behavioral deficits ceased soon after EGF was depleted from the pump [39], whereas in the neonatal model, the deficits persisted more than five months after EGF administration was completed at the neonatal stage [31].

A similar behavioral abnormality was also detected in the EGF-injection model of a nonhuman primate [44]. Apparent behavioral deficits such as stereotypic movement, vocalization, alert motion, and self-injury only emerged 5-6 years (i.e., monkey puberty) after EGF administration to a cynomolgus monkey neonate and were ameliorated by chronic treatment of the antipsychotic drug risperidone [44]. Therefore, the EGF-injection model may be established in a wide variety of animal species.

## 3. Life-Long Overexpression of EGF or NRG1 from Their Transgenes

In addition to the above injection models, we also analyzed the two types of transgenic (Tg) mice lines: type 1 neuregulin-1 overexpressing Tg mice and EGF-overexpressing Tg mice [45, 46]. In these transgenic lines, both the transgenes of EGF and NRG1 were expressed in the whole body, although their relative expression levels were higher in the central nervous system compared with those in the peripheral tissues.

EGF-Tg mice exhibited normal locomotion in the exploratory condition, and moderate deficits in context learning (Table 2). Sound startle responses of EGF-Tg mice were normal but their prepulse inhibition of startle responses was markedly lower than that of wild-type littermates [46]. In addition, EGF-Tg mice exhibited higher sensitivity to repeated cocaine administration in a locomotor test. Our preliminary studies indicate that social behavior scores of EGF-Tg mice appeared to diminish as well. Overall, EGF-Tg mice showed gross behavioral similarities to the EGF-injection model. In agreement to these behavioral traits, there was an enhancement in dopamine metabolism in the basal ganglia regions [46]. The results from EGF-Tg mice rule out the possibilities of experimental artifacts in the EGF-injection model; the behavioral deficits would be ascribed to any impurities in recombinant EGF samples or the production of anti-EGF antibody following EGF injections.

In our previous study, NRG1-Tg lines showed increased locomotor activity, a nonsignificant trend toward decreasing prepulse inhibition, and decreased context-dependent fear learning, but they exhibited normal levels of tone-dependent learning [45] (Table 2). In addition, our preliminary study indicates that social scores of both Tg lines were reduced. In contrast to the results from the NRG1-injection model, NRG1-Tg mice exhibited downregulation of dopamine metabolism in the corticolimbic regions. This reduction of dopaminergic phenotypes in NRG1-Tg mice had not been expected. The controversial results from the NRG1-Tg mice should be explored with respect to the neurotrophic activity of NRG1 on dopaminergic neurons [21, 38].

Some of the behavioral traits of the NRG1-Tg mice significantly resemble those of NRG1 knockout mice [see reviews; [47–53]]. Gene targeting of another ErbB1 ligand HB-EGF also generates the animal model for schizophrenia [50]. Although here we avoid to repeat the details of their behavioral phenotypes, it is noteworthy that both hypomorphic and hypermorphic expressions of the NRG1 or EGF-related gene produce several common behavioral phenotypes in mice. This commonality is quite surprising and raises a challenging question about the molecular and cellular mechanisms underlying the behavioral deficits induced by the opposite signals.

There is another fundamental question whether schizophrenia is associated with the upregulation or downregulation of the NRG1-ErbB4 signaling [5, 47]. A postmortem study indicates the upregulation of NRG1 expression [47], while an analysis of patients' blood suggests the downregulation of NRG1 expression that associates with schizophrenia and the risk SNP [5]. The clarification of this controversy should be the first step before arguing the pathologic implication of NRG1.

## 4. Neurobiological Underpinnings of the Behavioral Deficits Triggered by EGF and Neuregulin-1 Hypersignals

There is a large time gap between factor treatment (via injection) and the emergence of behavioral deficits in neonatal EGF- and NRG1-injection models [54–57]. Thus, we aimed to determine what kinds of signals from EGF or NGR1 contribute to the delayed emergence of the behavioral deficits at the postpubertal stages. In light of reported neurobiological activities of the ErbB ligands EGF and NRG1, we initially focused on the three neurotransmitter systems: GABAergic, glutamatergic (AMPA and NMDA receptors), and dopaminergic systems. The individual neuronal systems are composed of particular neurons expressing ErbB1 and/or ErbB4 and thus are reactive to EGF and NRG1.

When EGF was given to pups, GABAergic, glutamatergic, and dopaminergic markers were markedly affected in the acute phase. Specifically, GAD67, parvalbumin, GluA1 (AMPA-R), and GluN1 (NMDA-R) levels were influenced in the frontal cortex and/or midbrain of EGF-treated animals [17, 55, 56]. Dopamine synthesis, metabolism, and axon terminal arborization were shown to be markedly upregulated in most of the basal ganglia regions [20, 57]. In the postpubertal stages, however, most of these phenotypic influences became modest or undetectable, except for the presence of a hyperdopaminergic state in the globus pallidus [58]; tyrosine hydoxylase-positive fibers and varicosities were denser in the lateral regions of the globus pallidus of EGF-injected rats, compared with vehicle-injected rats.

The immediate influences of NRG1 injection in neonates were limited to dopaminergic and glutamatergic systems [24, 38]. Increases in dopamine synthesis, metabolism, and terminal arborization were found [38] as well as those in AMPA-type glutamate receptor levels [24] (Table 1). Although the acute effects on dopamine markers were similar to those seen in the EGF-injection model, those on AMPA receptors (GluA1) were opposite to that seen in EGF-injection models [17]. Again, the synaptic increase in AMPA receptor expression and function gradually diminished following the cessation of NRG1 injection in the postnatal stage [24].

As mentioned above, the NRG1-injection model also transiently exhibited gross abnormalities in the dopamine system. For instance, during postnatal NRG1-treatment, dopamine synthesis, metabolism, and axon terminal arborization were elevated in various brain regions including the basal ganglia and corticolimbic system [38]. The dopaminergic abnormalities in most of the brain regions were transient but those in the corticolimbic system continued until the postpubertal stages when the behavioral deficits emerged (Table 1). Therefore, we conclude that EGF-injection and NRG1-injection models share a persistent hyperdopaminergic abnormality; however, the target regions appear to differ between these models. The differential distributions of their receptors (ErbB1 and ErbB4) in the midbrain may illustrate the distinct influences of EGF and NRG1 on the dopamine system [15, 59]. Our quantitative study of* in situ* hybridization suggests that the EGF receptor (ErbB1) has a limited distribution in the nigrostriatal system and is less enriched in the ventral tegmental area (VTA)-corticolimbic system [20].

When we compared the behavioral and neurochemical phenotypes between the injection and TG models, we found significant differences (Tables 1 and 2): the magnitude of prepulse inhibition deficit was less pronounced or modest in NRG1-TG mice than in NRG1-injected mice. Moreover, context-dependent fear learning and exploratory motor activity deficits were prominent only in NRG1-TG mice. In this context, we suggest that spatial and temporal differences of NRG1 supply might alter target cell populations and their responses as various endophenotypes were seen among distinct NRG1-TG lines [45, 60–62].

## 5. Neurobehavioral Implication of a Pallidal Hyperdopaminergic State in the EGF-Injection Model

**Figure 5:**
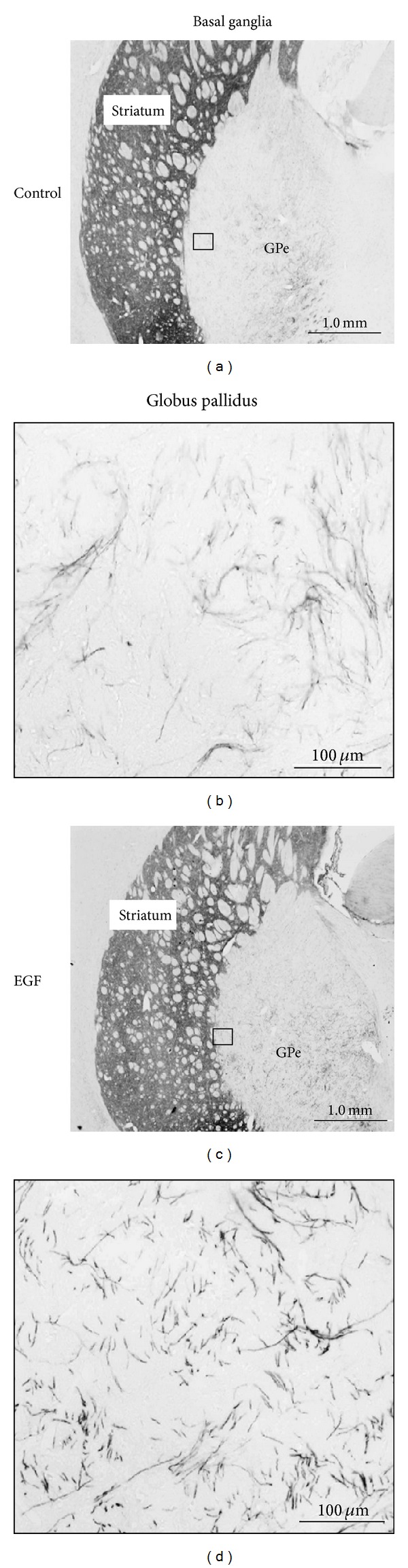
Neonatal EGF treatment enhances and maintains collateral sprouting of dopaminergic fibers in the globus pallidus (GPe). Neonatal rats were subcutaneously challenged with EGF (1 mg/kg body/day) for 9 days and grown until adulthood. EGF-treated and vehicle-treated rats were subjected to immunohistochemistry at the adult stage. In comparison with the tyrosine hydroxylase staining in the striatum (left), immunoreactivity in the lateral area of the GPe is elevated in the EGF-treated rats. Scale bars = 1000 and 100 *μ*m.

We further analyzed the EGF-injection model, focusing on the neuropathological abnormality of the dopaminergic system that continued over the postpubertal stage (Figure 5). The globus pallidus is implicated in the motor and cognitive regulation of the indirect pathway and is one of the major targets of the antipsychotics exhibiting D2 antagonism [63–65]. We found that more dense collaterals of nigrostriatal dopamine fibers innervate the globus pallidus in EGF-treated rats. Thus, we attempted to correlate any of the behavioral deficits of EGF-treated rats with pallidal dopamine dysfunction [58, 66].

**Figure 6:**
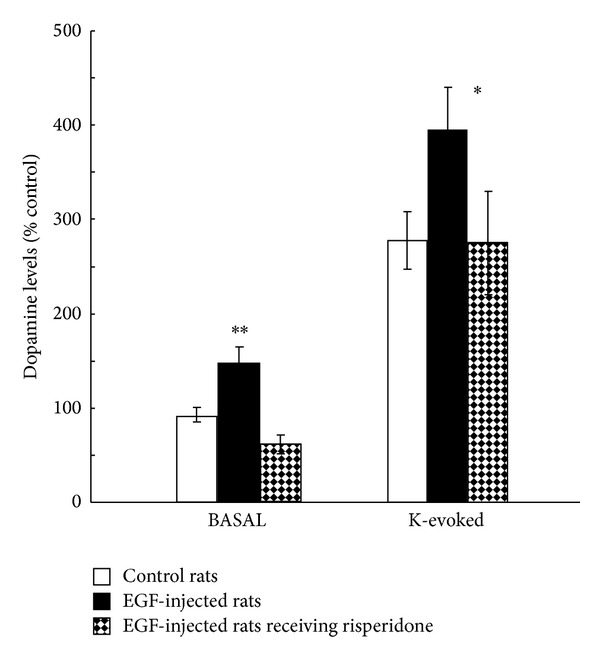
Local dopamine release from the globus pallidus was monitored by microdialysis. Dialysis probes were implanted in the globus pallidus of EGF-treated, vehicle-treated, and EGF-treated plus risperidone-medicated rats. Dopamine release of EGF-treated rats was elevated in both basal and high potassium (K) evoked conditions. These increases were normalized by subchronic treatment with the antipsychotic risperidone (1 mg/kg/day, 14 days total). *P < 0.05 and **P < 0.01.

In the globus pallidus of the EGF-injection model, there were persistent increases in tyrosine hydroxylase levels and dopamine content in the globus pallidus. Furthermore, pallidal dopamine release was also elevated in EGF-injected rats; however, the increased dopamine release was normalized by subchronic treatment with the antipsychotic drug risperidone (Figure 6). The amounts of pallidal dopamine release in individual animals were correlated with the magnitude of their prepulse inhibition levels. Single-unit recordings verified that the pallidal hyperdopaminergic state resulted in pallidal dysfunction with hyperactivation [66]. Corroborating these observations, the administration of dopamine D2-like receptor antagonists indeed ameliorated prepulse inhibition levels of EGF-treated rats as well as pallidal hyperactivity [58, 66]. Similarly, administration of ErbB inhibitors also normalized these behavioral impairments with their antidopaminergic actions in the globus pallidus [67].

Conversely, dopamine D2-like receptor agonist (quinpirole) administration to the pallidus of control rats induced prepulse inhibition deficits and pallidal frequent firing, confirming the pathophysiologic role of the pallidal hyperdopaminergic state [58, 66]. Impaired eye saccade, one of the common endophenotypes of schizophrenia patients, might reflect such a dopaminergic dysfunction of the indirect pathway in patients [68, 69].

## 6. Interactions of EGF-NRG1 Signals with Other Cytokines and Neurotransmitters

**Figure 7:**
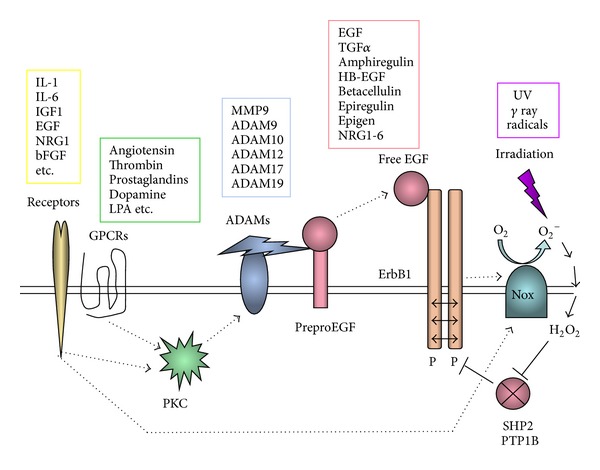
Ectodomain shedding of EGF-like precursors and transactivation of ErbB receptors. Cytokines (yellow box) and GPCR ligands (green box) activate metalloproteases in a disintegrin and metalloprotease (ADAM) family (blue box) via the activation of protein kinase C (PKC). These metalloproteases cleave the precursor proteins for EGF-like factors in the cell membrane and liberates the core EGF domain (red box). The soluble EGF-like factor diffuses into blood stream and acts on ErbB receptors. Alternatively, cytokine-triggered NADPH oxidase (NOX) activation results in the production of reactive oxygen species (ROS) and hydrogen peroxide, which inhibits the protein phosphatases (i.e., SHP2 or PTP1B) for ErbB kinases. UV or gamma ray irradiation (purple box) directly produces ROS and hydrogen peroxide. The attenuation of the phosphatases SHP2 (Src homology 2-containing protein tyrosine phosphatase) or PTP1B (protein tyrosine phosphatase 1B) markedly elevates basal phosphorylation (P) levels of ErbB receptors.

It is noteworthy that EGF-NRG1 signaling is secondarily evoked by other cytokines and neurotransmitters. This concept, namely, “ErbB transactivation,” has been well established in cancer biology and cell biology [70–73]. Inflammatory cytokines (IL-1, IL-6, TNF*α*) and their mediators of prostaglandins (PGEs) are potent transactivators of ErbB1 (EGF receptor) and trigger cell proliferation, leading to inflammation, wound healing or cancer priming. This process initiates with the protein kinase C activation, followed by ADAM (a disintegrin and metalloproteinase) activation (Figure 7). The activated ADAMs on cell surface shed (cleave) the membrane-linked precursor proteins of EGF, HB-EGF, and NRG1 (or their homologues) and liberate soluble EGF or NRG1, allowing them to bind to neighboring ErbB receptors or to diffuse into blood stream. Such transactivators for ErbB receptors now include GPCR agonists (angiotensin1, glutamate, dopamine, prostaglandins, thrombin, etc.), physicochemicals (UV light and ROS), growth factors, and cytokines (IGF1, EGF, NRG1, bFGF, IL-1, IL-6, etc.) [70–73]. Thus, dopamine itself is a potent activator for ErbBs and may provide positive feedback signals through the precursor shedding [74–76]. Accordingly, several other cytokines such as IL-6, which are implicated in maternal immune models for schizophrenia, potentially involve EGF-NRG1 signaling as well. IL-1, IL-6, and other inflammatory cytokines in the periphery can secondarily liberate EGF-like factors into the blood stream and may evoke ErbB signaling in various tissues including the brain.

## 7. Conclusion

**Figure 8:**
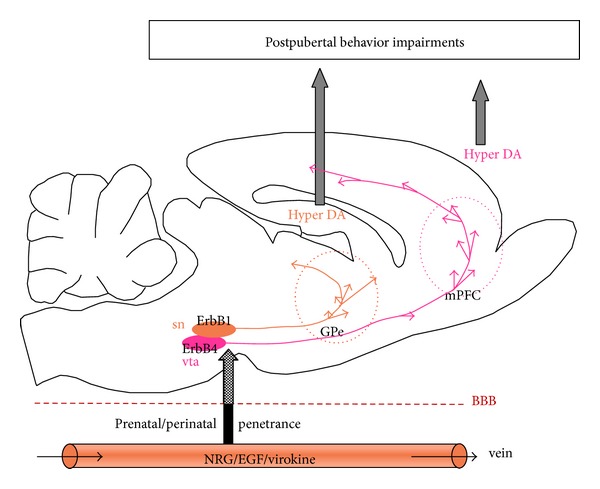
Ligands in the EGF family in circulation can penetrate the blood-brain barrier (BBB) during early development and act on the soma and terminals of dopamine neurons carrying ErbB1 and/or ErbB4. Exogenous EGF and NRG1 in the brain accelerate dopaminergic development and trigger ectopic hyperinnervation. The hyperdopaminergic innervation specifically persists in the globus pallidus and/or prefrontal cortex (mPFC). When the dopaminergic neurons are highly activated during and after adolescence, the excess amount of dopamine is released at the inappropriate sites, producing abnormal behavior and cognition.

The production of inflammatory cytokines and neurotrophic factors, such as EGF and NRG1, is regulated dynamically in the central nervous system as well as in the peripheral organs [10, 77, 78]. When the blood-brain barrier is not established, or when the blood-brain barrier is disrupted, these factors can efficiently reach brain neurons (Figure 8). In particular, EGF-like ErbB ligands are overproduced in the periphery or provided to blood stream following ischemic injury, inflammation, viral infection, and obstetric complications, which recruit inflammatory cytokines and trigger shedding and release of EGF-like precursors. For instance, peripherally produced EGF can penetrate the blood-brain barrier and act on immature nigral dopamine neurons, perturb their phenotypic development, and circuit connectivity in the basal ganglia, which presumably leads to life-long dysfunction [58, 64]. Alternatively, pox virus infection in fetuses or neonates may result in the production of NRG-like factors, which hamper the target connectivity of VTA dopaminegic neurons in the front-limbic regions [11].

In the postpubertal stage, when dopamine neurons are most highly activated at the basal state, the neurobehavioral consequences from the hyperdopaminergic dysfunction or ectopic innervation of dopaminergic terminals manifest (Figure 8). This cytokine-driven dopaminergic dysfunction might illustrate some of the psychopathological features of schizophrenia, although it is possible that the responsible factor(s) might be other cytokines other than EGF, NRG1, or virokine. In this context, the cytokine hypothesis for schizophrenia might occlude the other hypotheses such as the immunoinflammatory hypothesis, developmental hypothesis, and dopamine hypothesis.

## Figures and Tables

**Table 1:** Immediate and delayed effects of neonatal NRG1 and EGF administration.

		NRG1 Injection	EGF Injection
Behaviors	Sound startle	No change	No change
	Fear-learning	No change	No change
	Locomotor	No change	No change
	PPI	DECREASE	DECREASE
	Social behaviors	DECREASE	DECREASE

GABA	GAD 65/67	No change	(DECREASE)*
	PV	No change	(DECREASE)*

Glutamate	NR1 (GluN1)	No change	(DECREASE)*
	NR2 (GluN2)	No change	no change
	GluR1	(INCREASE)	(DECREASE)*

Dopamine	TH	INCREASE (cortex)	INCREASE (pallidus)
	DA	No change	INCREASE (pallidus)
	DOPAC	INCREASE (cortex)	INCREASE (pallidus)

*represent transient changes during neonatal administration. GAD: glutamate decarboxylase; PV: parvalbumin; TH: tyrosine hydroxylase; DA: dopamine; DOPAC: 3,4-dihydroxy-phenylacetic acid. Statistical significance represents *P* < 0.05.

**Table 2:** Behavioral and neurochemical effects of persistent NRG1 and EGF overexpression from their transgenes.

		NRG-1 TG	EGF-TG
Behaviors	Sound startle	No change	No change
	Fear-learning	INCREASE	Modest decrease
	Locomotor	INCREASE	No change
	PPI	Modest decrease	DECREASE
	Social behaviors	DECREASE	DECREASE

GABA	GAD 65/67	No change	No change
	PV	INCREASE (cortex)	No change

Glutamate	NR1 (GluN1)	No change	No change*
	NR2 (GluN2)	No change	No change*
	GluR1 (GluA1)	No change	No change*

Dopamine	TH	DECREASE (cortex)	DECREASE (striatum)
	DAT	No change	No change
	DA	DECREASE (hippocampus)	INCREASE (accumbens)
	DOPAC	DECREASE (hippocampus)	INCREASE (accumbens)

*represent effects on basal ganglia regions GAD: glutamate decarboxylase; PV: parvalbumin; TH: tyrosine hydroxylase; DA: dopamine; DAT: dopamine transporter; DOPAC: 3,4-dihydroxy-phenylacetic acid. Statistical significance represents *P* < 0.05 and “modest” indicates marginal changes with 0.05 < *P* < 0.10.
